# Cardiac magnetic resonance imaging in the evaluation of patients with hypertrophic cardiomyopathy

**DOI:** 10.21542/gcsp.2018.22

**Published:** 2018-08-12

**Authors:** Juan Carlos Brenes, Adelina Doltra, Susanna Prat

**Affiliations:** 1 Division of Cardiology, Mount Sinai Medical Center, Florida, USA; 2 Cardiology Department, Cardiovascular Institute, Hospital Clínic, Barcelona, Spain

## Introduction

Cardiac magnetic resonance imaging (CMR) is a useful technique in the evaluation of patients with suspected or diagnosed hypertrophic cardiomyopathy (HCM), and has an added value over other imaging modalities.

HCM is the most common genetic cardiomyopathy. Its prevalence is estimated by several global studies to be about one in 500 adults in the general population^1–5^. Over 1400 mutations in at least 11 genes encoding the cardiac sarcomere have been described^6^. HCM is morphologically characterized by primary hypertrophy of the myocardium, myocyte disarray and myocardial fibrosis^5,7^. Its presentation is highly heterogeneous and encompasses the entire spectrum range from an asymptomatic incidental diagnosis, to atrial and ventricular arrhythmias, to advanced heart failure or sudden cardiac death.

CMR can provide three-dimensional tomographic cardiac imaging with high spatial and temporal resolution, in any plane and without ionizing radiation. CMR has unique strengths which make it particularly well suited to provide detailed characterization of the HCM phenotype and, therefore, can aid in the diagnosis and potentially offer prognostic information^8^. In addition, CMR is the gold standard technique for quantification of ventricular volumes and function^9^.

**Table 1:** Added value of CMR in the evaluation of patients with increased left ventricular thickness.

**Morphological visualization**
Identification of the predominant morphologic phenotype
Quantification of maximum thickness
Differentiation between symmetric and asymmetric morphologies
Assessment of the segments involved
Evaluation of recesses, diverticula, aneurysms, clefts, and crypts
Structural evaluation of the mitral and aortic valve apparatus
Evaluation of the papillary muscles, and abnormalities of the tendinous chords
Differentiation of the true interventricular septum from other adjacent structures
**Functional evaluation**
Biventricular volume quantification and function
Assessment of global and segmental systolic thickening
Presence of dynamic left ventricular outflow tract (LVOT) and its cause
Valvular function
Perfusion
Strain
**Risk stratification**
Maximum thickness
Presence of LVOT obstruction
Presence of focal or diffuse fibrosis
Quantification of total ventricular mass and fibrosis
**Differential diagnosis**
Athlete’s heart
Hypertensive heart disease
Valvular heart disease
Other pathologies with LVOT obstruction: membrane or subaortic ring
Metabolic, deposit and infiltrative diseases
Tumours (intramyocardial fibroma)

A further advantage of CMR is its ability to characterize myocardial tissue. The evaluation of gadolinium retention by the myocardium in fibrotic areas has been extensively investigated and associated with clinical outcomes^10^. This has been improved with the recently developed T1 mapping techniques, which can provide information regarding diffuse fibrosis^11^.

Finally, CMR is also a useful tool for the differential diagnosis of HCM, being able to differentiate this disease from other conditions that present with increase in ventricular width, such as the physiologic changes associated to high performance athletes, hypertensive cardiomyopathy, aortic valvular disease and cardiac deposit diseases.

MR images are obtained by using the properties of the hydrogen nuclei (or protons) in an external magnetic field (i.e., the MR scanner), and applying short radiofrequency pulses. When radiofrequency pulses are applied, energy is transferred to protons. With pulse finalization, protons come back to their stationary equilibrium state, a process known as relaxation. The energy emitted during this relaxation state is used to generate the CMR image^12^. Fundamentally, there are two types of sequences in CMR. Spin echo (black-blood) sequences give information about anatomy and tissue characterization. Gradient echo (bright-blood) sequences, allow the visualization of cardiac movement (cine) and are used to analyze and quantify global and regional cardiac function. All other sequences used in CMR come from the modification of these two basic sequences.

## Indications and protocol in the evaluation of a patient with HCM using CMR

HCM is characterized by the increase in the diastolic maximum thickness of the left ventricle wall above the upper limit of normality in at least one segment, after the exclusion of other conditions that also present with hypertrophy. CMR has a high sensitivity and specificity to diagnose HCM and allows the detection of increase in wall thickness in areas where other techniques have limitations^7,13,14^. It can discriminate as well HCM from other conditions with phenotypic similarity. Hence, CMR is of great importance in the clinical, therapeutic and follow-up aspects of HCM. The added value of CMR in hypertrophic cardiomyopathy is summarized in Table 1.

### Advantages of CMR over echocardiography

Although transthoracic echocardiography (TTE) is a fundamental tool in the initial assessment of patients with left ventricular hypertrophy, the technique has some limitations, particularly in the presence of poor echocardiographic windows. Some heart segments (such as the LV apex) are especially difficult to image. Technical advantages of CMR in the evaluation of patients with HCM are listed in Table 2.

**Table 2:** Technical advantages of CMR in the evaluation of patients with HCM over echo.

TECHNICAL ADVANTAGES OF CMR
Accurate delimitation of the endocardial rim
Possibility of obtaining infinite imaging planes
Coverage of both ventricles
Better estimation of ventricular volumes and function
Better estimation of the magnitude of hypertrophy as compared to echocardiography
Better identification of focal forms
Visualize and quantification of fibrosis

When echocardiographic images are non-diagnostic, CMR has the distinct advantage of providing a high resolution imaging of the LV wall, allowing for accurate thickness measurements^15^. When comparing the two techniques, a significant difference in measurements has been reported, with a median difference between TTE and CMR SSFP imaging in the measured maximum wall thickness of 3 to 5 mm (being the maximum difference reported 17 mm)^16^.

**Figure 1.**
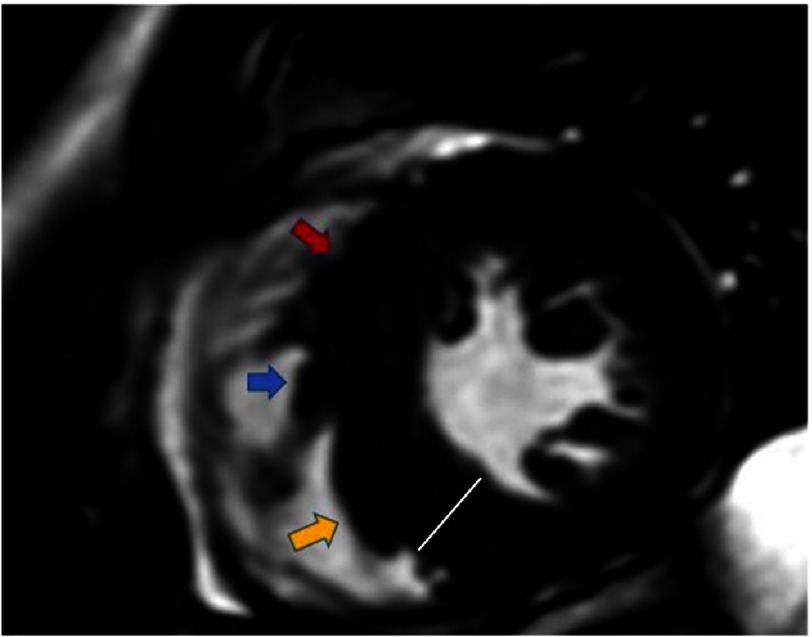
CMR short axis diastolic cine-SSFP imaging of a 19-year-old male athlete, referred for suspected HCM. Once the crista supraventricularis (red arrow), moderating band (blue arrow) and false tendons (yellow arrow) were appropriately identified, it was corroborated that the septal thickness was only slightly increased (12 mm, white line).

In addition to differences in measurement, CMR may identify areas of hypertrophy missed by TTE. This seems to be particularly concerning in the interventricular septum, medial anterolateral wall and in the apex, as reported by some authors, as these segments are particularly difficult to visualize by TTE^17,18^. The contrary case may also be true, as TTE may overestimate the true LV thickness in some patients due to the inability of TTE to accurately differentiate para-septal structures such as the origin of the right ventricle (RV) moderating band, false tendons and the crista supraventricularis. In those cases, the high myocardial tissue definition of CMR allows to properly differentiate those structures (Figure 1).

A subgroup of patients where CMR can be particularly useful is mutation carriers, as CMR can identify hypertrophic areas even in patients with a normal TTE. A report showed that penetrance in individuals carrying mutations that cause HCM was 70% when evaluated only with TTE, while it was close to 90% when using both TTE and CMR^19^. Therefore, CMR becomes even more relevant in young individuals with suspicion of HCM, hereditary family history, electrocardiographic abnormalities, inconclusive TTE or with other risk factors related to HCM.

### Standard CMR protocol in HCM

Due to its high contrast between the blood pool and the myocardium, steady-state free precession (SSFP) cine imaging is used for morphological assessment and to quantify ventricular volumes, ejection fraction, and mass, which has prognostic implications^14^. A complete set of SSFP slices acquired in the short axis plane from base to apex permits the visualization and measurement of hypertrophied regions, as well as quantify LV mass. SSFP images in standardized two, three and four-chamber planes provide additional morphological assessment. Cine SSFP can also demonstrate the presence of a turbulence jet across the left ventricular outflow tract (LVOT) in patients with obstructive HCM, and aid in the exact location of the flow obstruction site. Moreover, SSFP CMR can detect other abnormalities associated with HCM such as the presence of congenital ventricular outpouchings (recesses, diverticula, aneurysms, clefts, and crypts)^20^, anomalies in the mitral valve apparatus and abnormalities of the papillary muscles^21^. Late gadolinium enhancement (LGE) imaging provides non-invasive tissue characterization by identification of HCM associated interstitial and replacement fibrosis^22^. Phase contrast imaging (PC-CMR) or flow sequences can be used to quantify the outflow tract peak systolic velocity. Likewise, PC-CMR can be used to assess diastolic filling velocities, obtaining curves similar to those of echocardiographic Doppler.

Furthermore, there are additional sequences that may be useful in selected patients. T1-weighted multi-slice gradient-echo first pass gadolinium perfusion (either at rest or with stress medications) can be used to detect ischemic segments, either by an imbalance in blood supply in hypertrophied areas, or by associated atherosclerotic coronary disease or intramyocardial trajectories of the coronary arteries. Although still under investigation, the newly developed T1 mapping sequences can be used to detect diffuse fibrosis, which may go undetected on LGE imaging. Native and post-contrast T1 mapping has shown promise as a novel tool to support diagnostic, therapeutic and prognostic decision making^23,24^. Finally, strain analysis with tagging imaging can be useful in selected patients and settings, although is not routinely used in clinical practice, since it is time-consuming and requires specific software. With tagging sequences it is possible to evaluate the myocardial dynamic deformation during the cardiac cycle. A study found a reduced total systolic strain in septal and inferior regions in HCM patients, as well as reduced early-diastolic strain rates^25,26^. Recently, other techniques such as feature tracking have also been used for strain assessment.

Table 3 summarizes the mail CMR sequences used in the evaluation protocol of patients with HCM.

**Table 3:** Basic and advanced sequences of CMR in HCM.

**Basic sequences**	
Sequences	Objective
Cine SSFP sequences (4- 2- and 3-chamber views)	Structural assessment, motility evaluation
Cine SSFP short axis stack	Volume and functional evaluation
Cine SSFP sequences with orthogonal LVOT view	Rule out LVOT obstruction and SAM evaluation
Flow sequences LVOT (in-plane/through-plane)	LVOT obstruction presence and location. Peak velocity quantification (infraestimation as compared to echo)
Inversion-recovery fast gradient-echo (LGE assessment)	Presence and extension of fibrosis. Percentage of involvement in relation to the total myocardial mass.
**Advanced/optional sequences**	
Sequences	Objective
T1 mapping sequences pre- and post-contrast	Diffuse fibrosis and extracellular volume evaluation
Tagging sequences /Feature tracking	Dynamic myocardial deformation. Strain assessment
Perfusion sequences (rest/stress)	Detection of myocardial ischemia

## Role of CMR in the phenotypic classification of HCM

**Figure 2.**
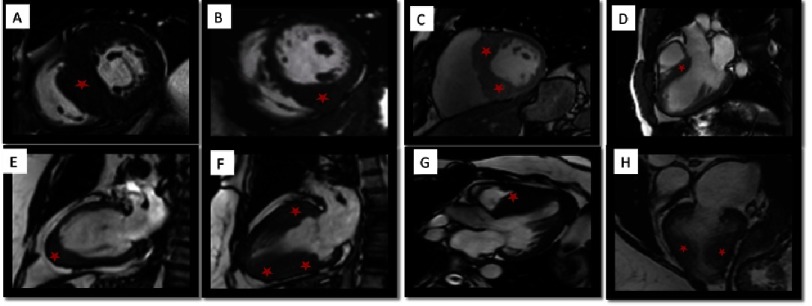
CMR imaging of different phenotypic expressions of HCM. A) Hypertrophic septal asymmetric cardiomyopathy. B) Asymmetric HCM with significant hypertrophy located exclusively in the inferior wall and in the lower portion of the interventricular septum. C) Asymmetric HCM with reversed septal contour. D) HCM with a focal hypertrophy in the basal segment of the anterior interventricular septum. E) Apical HCM. F) Diffuse HCM involving the anterior and inferior basal and middle walls. G) Asymmetric HCM with reversed septal contour. H) Mid-ventricular HCM with apical aneurism.

In HCM, a set of histopathological and anatomopathological conditions confer different phenotypes. CMR is a determinant tool that provides anatomical information and tissue myocardial characterization, allowing the proper grouping of these patients according to phenotype. The main indications of CMR in the study of patients with suspected or diagnosed HCM is summarized in Table 4. Figure 2 depicts the main HCM phenotypes.

**Table 4:** Indications for CMR in the study of patients with HCM.

Indications for the use of CMR
√ Inconclusive echocardiography/poor echocardiographic windows
√ Anatomical assessment, ventricular function quantification, and evaluation of fibrosis
√ Before surgical myomectomy
√ Patients with multi-level LV obstruction
√ Right ventricular (RV) outflow tract abnormalities
√ Apical involvement suspected (hypertrophy or aneurysm)
√ Arrhythmic risk stratification
√ Location of scarring and LV mass regression assessment after septal alcohol ablation or myectomy
√ Differential diagnosis (athlete’s heart, infiltrative cardiomyopathy)

### CMR in asymmetric septal hypertrophic cardiomyopathy

#### Sigmoid septal contour

**Figure 3.**
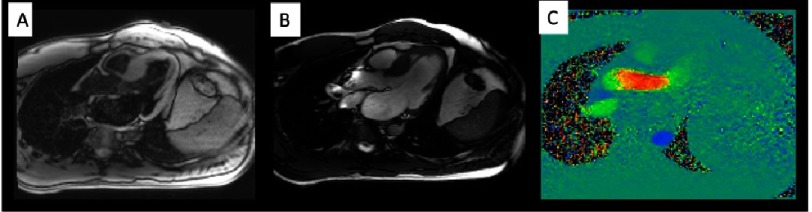
Asymmetric septal HCM with obstruction of the left ventricular outflow tract. A) Black blood (spin echo) 3-chamber view. B) SSFP-cine 3-chamber view. C) Phase-contrast sequence demonstrating flow acceleration in the LVOT (orange coloured spectrum).

It is the most common form of HCM described in the literature, representing up to 70% of all cases^3^. In this particular phenotype, the interventricular septum acquires a sigmoid morphology as visualized by cine SSFP sequence, being the basal anterior interventricular septum the segment most frequently affected^13^; in order to ensure a correct thickness assessment, the adjacent parietal muscle band of the right ventricle should be excluded. This form of HCM is frequently associated with LVOT obstruction and with anterior systolic movement of the mitral valve (SAM). In addition to LVOT, flow obstruction can additionally occur at different levels of the ventricular cavity (such as the mid inferior segment) or it can be associated with anomalies of the mitral valve apparatus and the arrangement of the papillary muscles. In a study by Maron et al, up to 34% of patients with this phenotypic variant had mitral valves greater than two standard deviations compared to controls, independently of the degree of hypertrophy, age and the presence of obstruction^27^ (Figure 3).

The second most frequent form of asymmetric septal HCM is the hypertrophy of medial inferior interventricular septum^28^.

The most frequent form of LGE found in the sigmoid septal phenotypic variant is the presence of myocardial gadolinium retention in the RV septal insertion points. Finally, as a similar hypertrophy pattern can occur in other cardiac pathologies, is particularly important in this HCM variant to exclude other causes of LVOT obstruction (see differential diagnosis).

#### CMR in HCM with reverse septal contour

**Figure 4.**
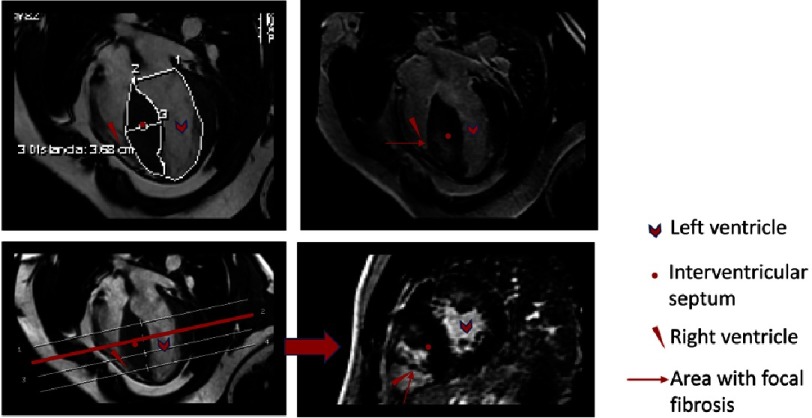
44 year-old female with asymmetric septal HCM, with reversed septal contour in a C-shape and extensive intramyocardial fibrosis.

In this phenotype the septal thickening occurs predominantly towards the middle segments of the LV. The septum, then, acquires a C-shape morphology, which is usually not accompanied by LVOT obstruction, but may be associated with medial ventricular cavity occlusion. Fibrosis is usually observed in the areas of major hypertrophy, being described as a “patchy” or “cotton-like” pattern^29^ (Figure 4).

#### CMR in HCM with associated midventricular obstruction and apical aneurysm

**Figure 5.**
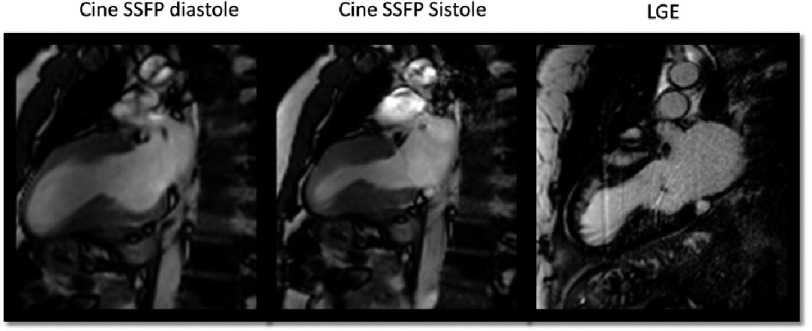
64 year-old patient with asymmetric mid-ventricular HCM (2-chamber view).

In this asymmetric variant there is marked mid-ventricular septal hypertrophy along with a significant decrease in ventricular volumes due to narrowing of the cavity. The apical region in this phenotype may be dilated, with a characteristic “hourglass” shape (Figure 5). A percentage of these patients develop fibrosis in apical segments due to intramural or subendocardial vascular involvement, related to the significant increase in intraventricular pressure^30^. This phenotype must be differentiated from other forms of apical presentation.

#### CMR in Apical Hypertrophic Cardiomyopathy (AHCM)

AHCM is characterized by hypertrophy of the apical myocardium with a spade-like deformity of the LV cavity. Due to window limitations when imaging the LV apex, CMR imaging can identify more easily this HCM variant as compared to TTE^28,31^.

Typically, this variant is characterized by an obliteration of the cavity in the apical region, with a typical image of “sword-tip” in 2-chamber view. The apex’s wall width is usually thicker than 15 mm and the ratio between the width of the apical and the basal segments is more than 1,5^28,31,32^.

Although AHCM is reported to have a better long-term prognosis than other forms of HCM, a potential for arrhythmic events still exists, and a third of patients can present with ventricular tachyarrhythmia^31,33^.

A study found a significant association between intramural extensive LGE and ventricular tachyarrhythmia, even in cases of AHCM^33^.

#### CMR in symmetric HCM

In this phenotype, a concentric and diffuse pattern of LV hypertrophy is present, which results in the reduction of ventricular volumes^3,15^. The prevalence of this particular phenotype can be as high as 40% of cases of HCM.

Before confirming the diagnosis, two important points must be considered. First, appropriate differential diagnosis must be made with other entities that present with diffuse hypertrophy of LV myocardium, such as hypertensive cardiomyopathy, amyloidosis, Fabry disease and athlete’s heart. Secondly, a diffuse hypertrophy can be the consequence of a severe increase in overload due to a significant obstruction of the LVOT; such obstruction can occur in diverse levels of the septum. In both cases, the use of RMC is determinant^13,34^.

#### CMR in focal HCM

A minor percentage of patients show an increase in ventricular width limited to small and focal areas of the ventricle, usually confined to one or two segments. This can also be the clinical presentation of early stages of HCM.

Focal HCM is most common in the basal segments of the septal, anterior and anterolateral walls of the LV, being the septum the most frequently affected. Due to its focal nature, the indexed total ventricular mass in this phenotype is usually normal^35^.

Focal HCM is a controversial subject in the elderly population as, when cardiac hypertrophy is found, itis commonly due to overload increase such hypertensive states, myocardium remodeling and aging, and is not necessarily related to genetic anomalies^5^. Therefore, a differential diagnosis should be stablished between these entities and HCM.

#### CMR in right ventricle (RV) involvement in HCM

In a third of patients with HCM, a hypertrophied RV can be observed, most commonly near the insertion of the RV wall into the septum. Although the prognosis entailed by morphologic changes in the RV is yet unknown, CMR, as the technique of choice for RV assessment, plays a fundamental role in the detection of this phenotypic expression^36,37^.

In addition to detect RV hypertrophy, CMR is also able to identify other abnormalities, such as the presence of prominent RV muscle structures like the crista supraventricularis (Figure 1).

**Figure 6.**
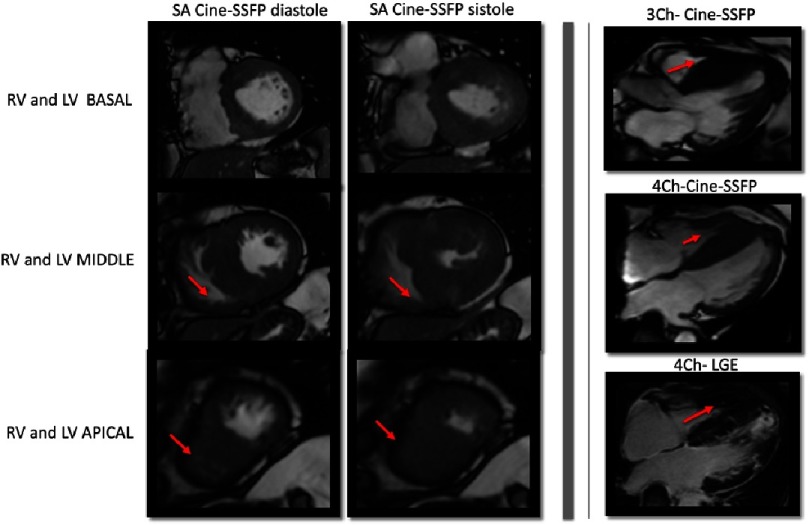
Hypertrophic septal asymmetric cardiomyopathy with apical obliteration and obliteration of the right ventricle (red arrows).

Similarly, in patients with subpulmonic RV obstruction due to RV free wall hypertrophy and secondary RV outflow tract narrowing, CMR can characterize the precise location and extent of the hypertrophy (Figure 6).

#### CMR and end-stage HCM

HCM usually shows normal or supernormal systolic function of the LV. However, 5 to 10% of patients with HCM have an LV ejection fraction below 50%, which is referred to as “end-stage”, “dilated phase”, or “burn-out” HCM^35^. These patients have a poor 5-year prognosis due to sudden cardiac death (SCD) and progressive heart failure (HF)^38^.

CMR can measure the ejection fraction accurately as well as identify extensive myocardial scarring in end-stage HCM. A study found that LGE extent was significantly larger in dilated end-stage HCM patients which was a significant predictor of poor outcome^38^.

#### Evaluation of anomalies of the mitral valve apparatus and papillary muscles

Over one third of HCM patients may present with substantially elongated anterior or posterior mitral valve leaflets^27^. These morphologic valvular abnormalities likely represent a primary phenotypic expression of HCM^21^. Elongated mitral valve leaflets also contribute substantially to increased subaortic gradients, particularly in those HCM patients in whom the mitral leaflet length exceeds 2-fold the transverse dimension of the outflow tract at end-systole^39^.

CMR has also expanded our knowledge of other morphologic abnormalities in patients with HCM. HCM patients frequently have an increase in the number of papillary muscles, including 3 or 4 papillary muscles in almost half of the patients according to some series^21,39^, while hypertrophy of the papillary muscles is also common. Furthermore, there appears to be a subgroup of HCM patients with normal total LV mass, who show substantially hypertrophied papillary muscles^21^. In such patients, the cardiomyopathic process either disproportionally involves the papillary muscles, or preferentially affects them.

## Risk stratification and prognosis

### Evaluation of maximum thickness and ventricular mass

Non-invasive imaging of LV wall thickness has proven to have a role in risk stratification: LV hypertrophy of ≥ 30 mm identifies HCM patients at high risk of arrhythmia who could benefit from ICD therapy for SCD prevention^1,2^. Therefore, accurate assessment of maximal wall thickness is an essential part of the initial evaluation of all HCM patients. Previous observations have demonstrated that CMR can identify massive LV wall thickening (≥ 30 mm) that was underestimated in TTE^16^. Due to the variable distribution of LV hypertrophy, CMR-derived LV mass provides the most accurate assessment of the overall extent of LV hypertrophy in this disease^1^. As a result, LV mass may represent a marker for adverse risk and could be helpful for risk stratification. However, long-term prospective CMR studies are needed before establishing the precise relationship between LV mass and outcome.

### Late gadolinium enhancement

**Figure 7.**
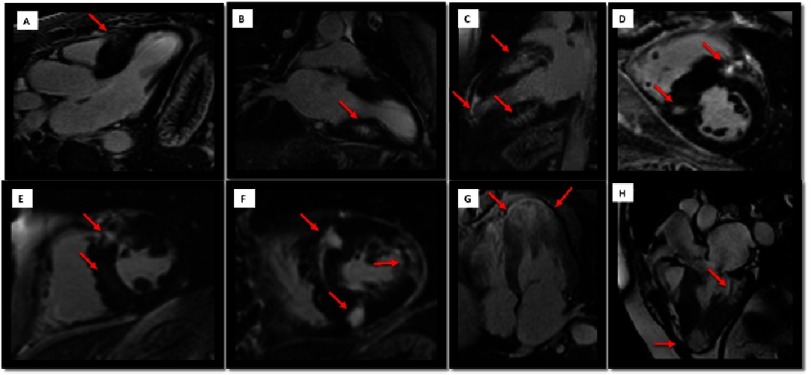
Examples of LGE patterns (red arrows) of patients with HCM. A) Phenotype limited to the anterior interventricular septum, where a discrete patchy pattern of localized fibrosis is observed. B) Hypertrophy of the basal and medial segments with mesocardial fibrosis localized in the inferior wall. C) Diffuse involvement and significant mesocardial fibrosis in the areas of greater hypertrophy. In addition, important thinning and fibrosis in the apical level is observed. D) HCM with intense focal fibrosis at the level of the right ventricular insertion points in the interventricular septum. E) Almost transmural LGE at the level of the mid-anterior interventricular septum and very tenuous inferoseptal mesocardial enhancement. F) Marked mesocardial septal LGE and at the insertion points, as well as quasi-transmural mesocardial LGE in the lateral wall. G) Diffuse LGE in a patient with severe apical HCM, with an uneven distribution pattern. H) LGE with a subendocardial pattern in the inferolateral basal segment with wall thinning. Fibrosis is also observed in an apical aneurysm.

The LGE technique is the only non-invasive imaging method that can detect the presence and extension of localized myocardial fibrosis that, in its turn, has been proposed as the substrate for arrhythmias and heart failure^40^. Furthermore, LGE may be associated with increased myocardial stiffness and adverse LV remodeling. In adult patients with HCM, LGE can be present in 60 to 70% of the individuals^13,22,41^. Regarding children, a study demonstrated LGE in 46% of children and adolescents with phenotypic HCM, with a 2.4 g increase of LGE per year^42^. As it has already been discussed when describing HCM phenotypes, LGE patterns in HCM patients may vary greatly, and a wide range of patterns, locations and distribution of LGE have been described (Figure 7).

The most common pattern (in approximately 30% of patients) is patchy and mesocardiac, being the septum and the LV free wall the most commonly affected areas^43^. Other less common locations are the isolated involvement of the lateral wall, apex, septum, papillary muscles, or the insertion points of the right ventricle in the LV septum. As previously described, some LGE patterns are more common in certain phenotypic expressions of HCM.

In some patients the LGE pattern may simulate coronary distribution, and transmural extent of LGE may be present in one-half of HCM patients^44^. Patients with LGE have greater maximal LV wall thickness and LV mass index than patients without LGE^40,43^. On an individual patient basis, a relationship is also present between segmental LV wall thickness and LGE.

Although LGE is usually present in segments with hypertrophy, in some end-stage cases some segments may appear thinned with transmural fibrosis^38^.

**Figure 8.**
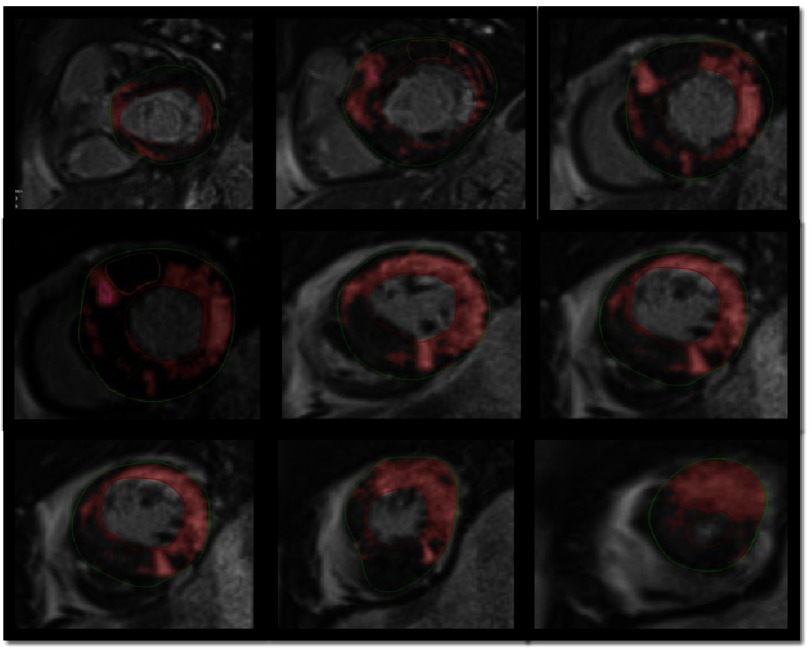
Semi-automatic quantification of fibrosis using short-axis slices from base to apex, selecting 2 standard deviations of signal intensity to define LGE (pink zones). Fibrosis quantification: 32% of the total myocardial mass.

The extent of LGE can be quantified (sum of the enhanced areas measured in grams) or expressed as a proportion of the total left ventricular mass (percentage of late gadolinium enhancement) (Figure 8). The percentage of fibrosis varies substantially according to the quantification method used. From those methods, the only validated method against necropsy is the semi-automatic 2-standard-deviation technique, which consists in defining LGE as a 2-standard deviation above the mean signal intensity of the distant myocardium, and constitutes the preferred quantification method^45^.

LGE is hardly ever observed in mutation carriers without LVH. In a study including patients with pathogenic sarcomere mutations and hypertrophic cardiomyopathy, subjects with mutations but no LV hypertrophy, and controls, CMR studies showed LGE in 71% of subjects with overt hypertrophy but in none of the mutation carriers without hypertrophy^46^.

Different studies have published an increase in the risk of ventricular arrhythmias in patients with HCM related to the presence of fibrosis evaluated by LGE in comparison with individuals without LGE^10,47–50^.

Another report described that a myocardial scar mass of more than 7 g on LGE predicted the risk of developing ventricular tachycardia with 75% sensitivity and 82% specificity^51^. Kwon et al. using Holter monitoring reported an increase in the presence of arrhythmias in patients with myocardial fibrosis documented by the presence of LGE^33^. Several studies with different scanning protocols and methods for LGE quantification have investigated the association with LGE and sudden cardiac death (SCD)^49,50,52–58^. Table 5 summarizes the main findings. Although a consistent association between SCD and LGE was demonstrated, a meta-analysis published in 2012 shed some doubt on these results. In this meta-analysis, it was concluded that a significant relationship exists between LGE and cardiovascular and all-cause mortality in HCM, but only a trend towards an increased risk of Sudden Cardiac Death (SCD) was observed^59^.

**Table 5:** Main studies that compared the association of LGE and sudden cardiac death.

Study, year (reference)	Center location of the study/Design	N	Follow up (years)	Sudden cardiac death rate	
				LGE +	LGE −
Maron, 2008^52^	USA/Prospective	202	1,9	3,60%	3,29%
Bruder, 2010^49^	Germany and USA/Prospective	220	3,0	6,75%	1,38%
O’Hanlon, 2010^50^	UK/Prospective	217	3,1	3,67%	1,23%
Rubinshtein, 2010^53^	USA/Retrospective	424	3,6	3,34%	0%
Hen, 2014^55^	Japan/ Retrospective	345	1,8	1,19%	0%
Smith, 2014^54^	USA/Retrospective	30	2,3	5,8%	0%
Ismail, 2014^56^	UK /Prospective	711	3,5	4,31%	1,66%
Chan, 2015^58^	USA/Prospective	1293	3,4	2,98%	0,94%
Klopotowski, 2016^57^	Poland/Prospective	328	3,1	6,19%	0%

Derived from this data, the 2014 European Guidelines^1^ mention that published studies are limited by selection and referral bias, incomplete risk assessment and differences in scanning and postprocessing protocols and, although the extent of LGE on CMR may be useful in predicting cardiovascular mortality, the available data did not support the use of LGE to predict SCD.

Another three meta-analysis have intended to investigate this issue^60–62^. The most recent meta-analysis on the topic, with a sample of 1734 patients with LGE and 2036 without, concluded that LGE is significantly associated with SCD/aborted SCD risk, all cardiac death and all-cause mortality in patients with HCM^62^.

Therefore, the latest data suggests that future risk stratification scales should probably include LGE evaluation for prediction of SCD, although it is not yet included in the guidelines. Table 6 summarizes the main systematic reviews, based on these conclusions it is possible to make this assertion.

**Table 6:** Main systematic reviews and meta-analysis that evaluate the relationship between CMR- late gadolinium enhancement and clinical outcomes.

Author	Journal	Year	Patients		Average follow-up (years)	Main conclusion
			With LGE	Without LGE		
He D et al^62^	Heart Lung	2018	1734	2036	2,9	LGE is significantly associated with SCD/aborted SCD risk
Weng Z et al^61^	JACC Cardiovascular Imaging	2016	1658	1335	3,06	Quantitative LGE by CMR exhibited a substantial prognostic value in SCD events prediction
Briasoulis et al^60^	Heart	2015	1653	1414	3,05	LGE is significantly associated with SCD risk
Green et al^59^	JACC Cardiovascular Imaging	2012	638	426	3,1	Significant relationship between LGE and cardiovascular mortality, heart failure, death
						LGE and SCD/aborted SCD displayed a trend toward significance

**Figure 9.**
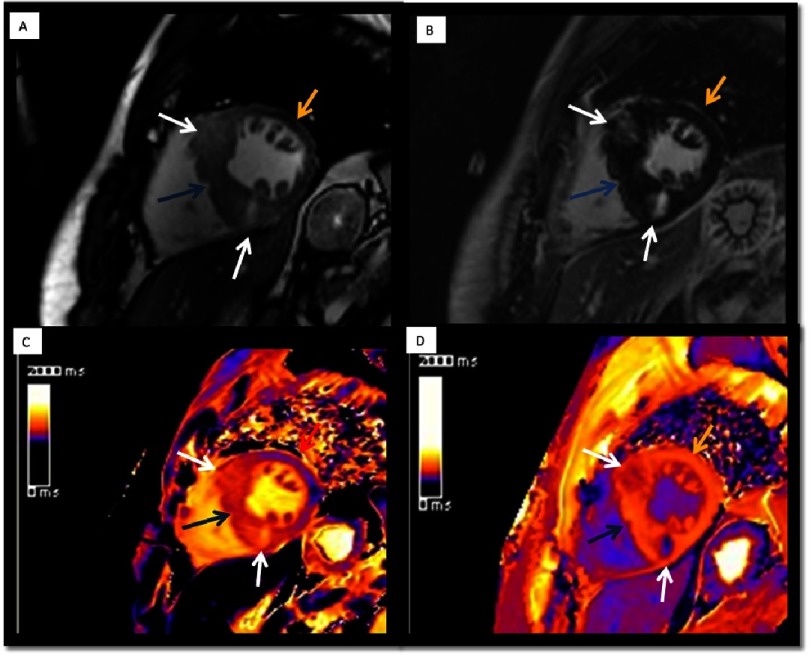
Patient with asymmetric septal HCM. LGE and T1 mapping sequences were used to assess the presence of focal (arrows) and diffuse fibrosis. A) SSFP-cine-short axis view in contrast CMR. B) LGE-sequence-short axis view that shows the enhacement in the insertion points of the RV in IVS. C) T1 mapping sequence without contrast. T1 native in the focal fibrosis zone (white arrows) was 1240 ms (normal between 950 to 1050 ms). In the IVS but no focal fibrosis (gray arrow): 1126 ms and in non-hypertrophic zones: 1035 ms (blue arrow). D) T1 mapping sequence after contrast infusion. Shorter times in focal fibrosis zones.

### T1 mapping in the evaluation of diffuse fibrosis and extracellular volume

T1 mapping measures the longitudinal relaxation time or T1, which is the time it takes for the protons to re-equilibrate their spins after being excited by a radiofrequency pulse. T1 varies in different tissues and may change in pathologic conditions. T1 mapping sequences permit the quantification of native T1 (assessing both intracellular and extracellular compartments), T1 after gadolinium contrast administration (reflecting only the extracellular compartment) and, with those two values and after correcting for the hematocrit, extracellular volume (ECV), which represents the percentage of myocardial tissue not occupied by cells (i.e., the extracellular space). Normal ECV values of 25.3 ±3.5 % have been reported in healthy subjects at 1.5 Tesla CMR^11^.

As discussed earlier, LGE is the reference standard for non-invasive imaging of myocardial scar and focal fibrosis. Diffuse fibrosis, however, may go undetected on LGE imaging. As diffuse fibrosis is also present in HCM, native and post-contrast T1 mapping have shown promise as a novel biomarker (Figure 9). Native T1 values are prolonged in HCM and correlate with wall thickness. Also, native T1 can be used to differentiate HCM from other forms of cardiac hypertrophy, such as hypertensive cardiomyopathy^63^, Fabry’s disease^64^, or amyloidosis^65^. In addition, some initial data suggest that T1 values could be used to differentiate control patients from those with a positive genotype for HCM and a negative phenotype, although this warrants confirmation in further studies^66^. These initial reports suggest that T1 mapping may be useful for risk stratification in HCM, but, up to now, no clear evidence exists.

Further works have used T1-mapping derived parameters after contrast administration (post-contrast T1 and ECV). Post-contrast T1 has been found to be reduced outside areas of LGE, whereas ECV in those areas has shown to be in the upper normal range of normal patients^23^.

Findings from the International T1 Multicenter Cardiovascular Magnetic Resonance Study showed that native T1 and ECV were significantly higher in HCM compared with hypertensive patients, even when including HCM patients without LGE and hypertensive subjects with LV wall thickness of >15 mm^63^. Some initial data also suggests that T1 mapping before and after gadolinium injection differs between HCM and athlete’s heart^67^. However, more information is needed regarding the cut-off values that effectively differentiate these two entities.

Regarding the pediatric population, a small study including 21 HCM patients and 21 controls found that hypertrophied myocardium had significantly higher native T1 and ECV compared to non-hypertrophied myocardium in HCM^68^. This has also been demonstrated in another small study in an adult population, which also found elevated T1 time in 30% of LGE negative segments of HCM individuals^69^.

Regarding the usefulness of T1 mapping techniques for risk stratification, only very few evidence exist. One of the few studies on the topic concluded that the combined use of the SCD risk score and global ECV could potentially improve selection of HCM patients for ICD implantation^70^.

### Evaluation of left ventricular outflow tract (LVOT) obstruction

**Figure 10.**
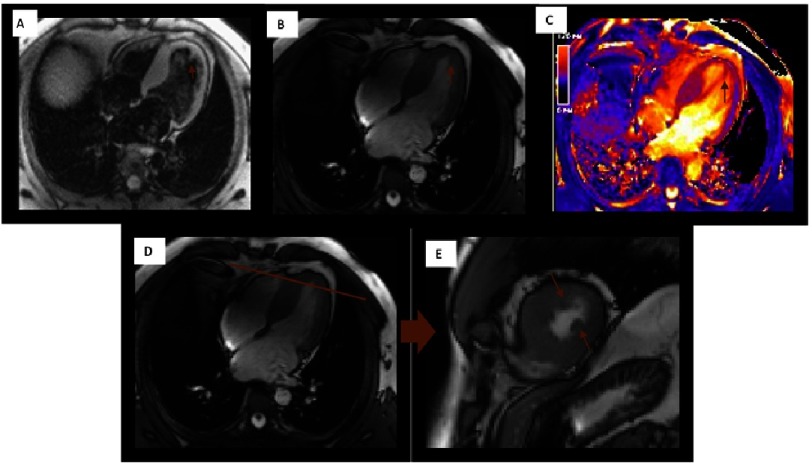
46 year-old patient with obstructive HCM and apical implantation of the papillary muscles. A) Black blood (spin echo) 4-chamber view. B) SSFP-cine 4-chamber view. C) T1 mapping sequence showing the implant site of the tendinous cord. D) and E) Long axis and corresponding short axis view, in which the abnormal implantation of these muscle can be observed.

Mechanical obstruction of the LVOT due to systolic anterior motion of the mitral valve (SAM) is one of the causes leading to exercise intolerance and heart failure in HCM. Detecting the presence and degree of LVOT obstruction influences treatment, as invasive measures (such as myectomy or alcohol septal ablation) should be considered if the obstruction is non-responsive to pharmacological treatment. CMR flow sequences can locate the site of flow obstruction and identify anomalies contributing to outflow obstruction, such as anomalous insertion of the anterior papillary muscle (Figure 10) or elongated mitral valve leaflet^71^.

### Left atrial structure and function evaluation in HCM

**Figure 11.**
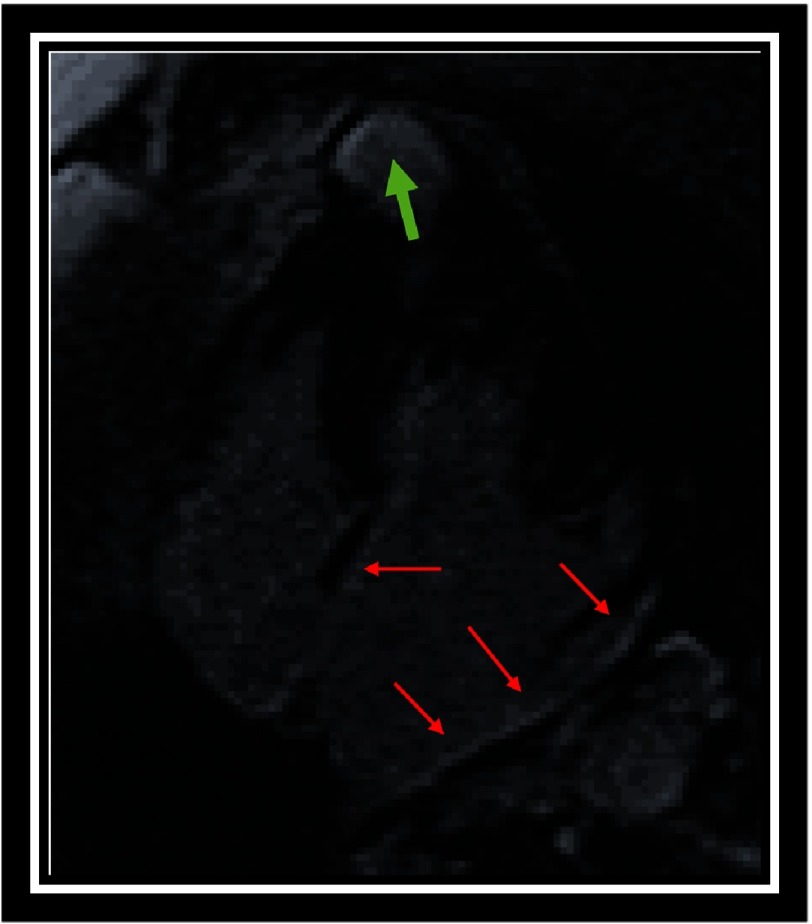
LGE in a patient with end-stage mid-ventricular HCM. Left atrial fibrosis (red arrows) and fibrosis located in an apical aneurysm (green arrow) can be observed.

Alterations in the structure and function of the left atrium have been evaluated in different studies as predictors of adverse cardiac events and arrhythmias. Maron et al published a prospective study with 427 patients, where it was shown that an atrial volume greater than 118 mL or an ejection fraction of the left atrium less than 38% may be predictive factors for the development of AF in patients with HCM and sinus rhythm^72^ (Figure 11).

### Evaluation of diastolic dysfunction by CMR

It is a common feature of HCM and usually evaluated with echocardiography. Therefore, very few data on CMR is available. Some initial data suggest that the presence of LGE at right ventricular insertion points is correlated with increased estimated LV filling pressure^73^.

### The role of CMR feature tracking and strain estimation in HCM

**Figure 12.**
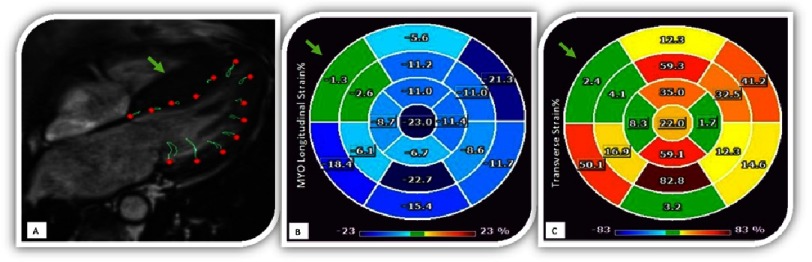
Evaluation of longitudinal and transverse myocardial strain using feature tracking in a patient with HCM. A) Cine-SSFP 4-chamber view of a patient with predominantly septal hypertrophy (green arrow). B) Longitudinal strain obtained from cine-SSFP in 3-chamber, 2-chamber and 4-chamber views. Longitudinal strain is expressed in percentage as a negative value, the myocardial deformation is lower as the value approaches 0. Note the significant decrease in the septal deformation (green arrow). C) Transverse strain obtained from cine-SSFP in short axis basal, middle and apical slices. Transverse strain is expressed in percentage as a positive value, transverse myocardial deformation is lower as the value approaches 0. Note in this case also the significant decrease in septal deformation (green arrow).

Echocardiographic studies suggest that strain imaging can help in the diagnostic evaluation and can offer some prognostic information. Some evidence exists regarding the use of CMR for strain analysis in HCM patients. In a study using the feature tracking technique (FT) it was demonstrated that LV mass, LV thickness, and LGE are independent contributors to reduced global LV strain assessed by CMR-FT^74^. This paper also suggests that reduced myocardial strain is associated with cardiovascular mortality and heart failure, although it is important to note that the event rate was low. Further research should confirm those findings in a larger population. Although good intra- and interobserver agreements have been reported for FT^75^, it must be noted that this technique suffers from inter-vendor variability, which should be taken into account (Figure 12).

**Figure 13.**
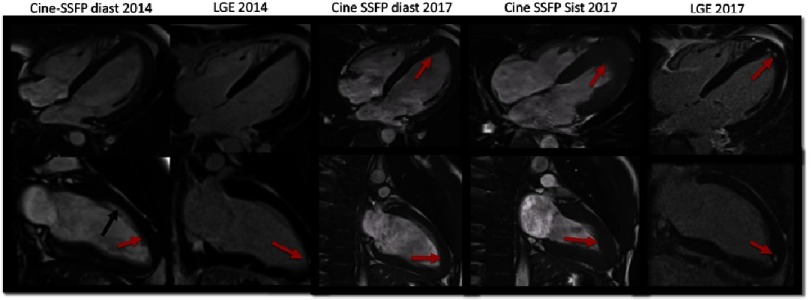
24 year-old athlete with T wave inversion in the ECG. In the initial evaluation, the echo was normal and CMR did not show any relevant findings. In a second examination three years later, an apical hypertrophic cardiomyopathy was diagnosed with CMR, with presence of focal fibrosis in that area.

## Differential diagnosis of conditions that present with an increase width of the ventricular wall

### Athlete’s heart

Physical training is associated with adaptation changes of the heart, including an increase in ventricular width. On the other hand, HCM is one of the main causes of SCD in the young. A cross-over phenotype exists between HCM and athlete’s heart, that consists in light non-symmetric cardiac hypertrophy^76^. Therefore, distinguishing athlete’s heart from HCM is determinant and often a diagnostic challenge.

Petersen *et al* reported that adaptive changes of the heart in athletes can be distinguished appropriately from pathologic forms of cardiac hypertrophy using CMR. In particular, a diastolic wall-to-indexed volume ratio <0.15 mm/m2/ml differentiates athlete’s heart from other factors of hypertrophy (HCM, hypertension and aortic stenosis) with a 99% specificity^77^.

In addition to an accurate measurement of LV width and volumes, CMR can also detect other myocardial anomalies such as multiple clefts, focal patterns of hypertrophy, and fibrosis, which would support the diagnosis of HCM (Figure 13). It is important to point out that the absence of LGE does not necessarily rule out HCM, as up to 50% of young athletes with a suspected HCM or positive genetic testing may not present LGE in initial stages. It has already been discussed that diffuse fibrosis assessment may play a role in the differential diagnosis^34^.

Lastly, in the presence of ECG abnormalities, ventricular arrhythmias or premature frequent heartbeats in an athlete, CMR can be useful to detect not only HCM, but also other conditions such as arrythmogenic cardiomyopathy and myocarditis.

### Hypertensive heart disease

Long-standing hypertension results in a usually concentric hypertrophy which can be more pronounced in the basal septum (“septal bulge”). An asymmetric pattern of hypertrophy favors a diagnosis of HCM over hypertensive cardiomyopathy, although some patients with HCM may present with a symmetrical pattern of hypertrophy. Additionally, presence of LV outflow obstruction due to SAM favours HCM over hypertensive cardiomyopathy^78^. It has also been mentioned that T1 mapping techniques may help in the differential diagnosis^63^, although this requires validation in future studies.

### Infiltrative cardiomyopathies

**Figure 14.**
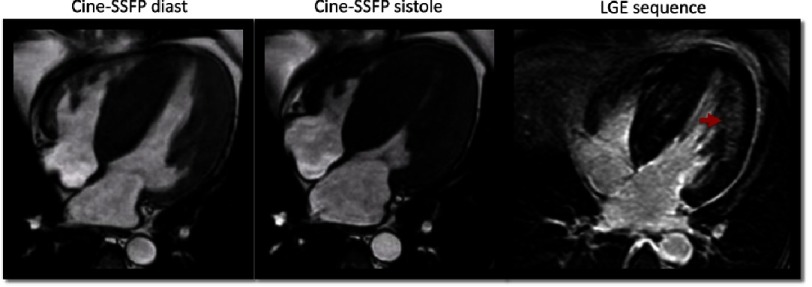
55 year-old with an echo-based diagnosis of HCM. CMR imaging shows diffuse hypertrophy, lateral wall LGE, and low T1 values in the septum. These findings were compatible with Fabry’s disease. The diagnosis was confirmed with laboratory testing.

This spectrum of diseases includes an increase in wall thickness as part of their phenotypic expression. The age of presentation and non-cardiac manifestations can be helpful in the diagnosis, although in some cases the heart may be the initial form of presentation. In amyloidosis, a typical LGE pattern characterized by global subendocardial LGE and impossibility to null the normal myocardium can be seen, together with very high native T1 and ECV values^65^. Fabry’s disease usually presents with hypertrophy in the septum and lateral wall with LGE confined to the basal inferolateral wall (Figure 14); also, lower than normal T1 values have been reported^64^. Danon’s disease presents with marked hypertrophy and extensive LGE^79^.

### Other conditions

Valvular heart disease such as aortic valve stenosis or subaortic obstruction usually are associated with asymmetric hypertrophy. CMR may identify the underlying lesion and contribute to its functional assessment.

**Figure 15.**
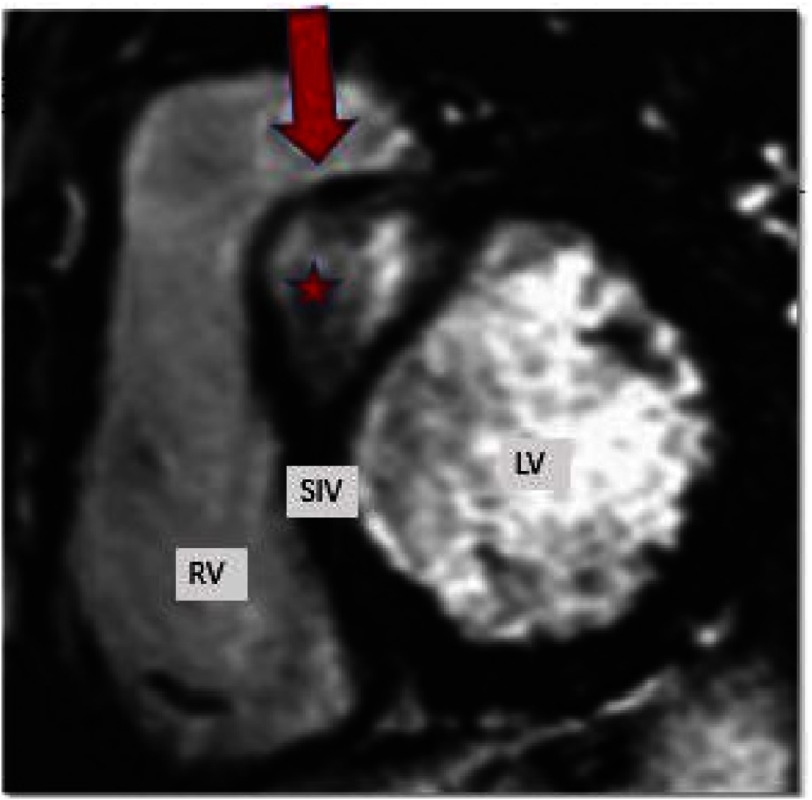
LGE imaging of a patient with a myocardial fibroma (red star). Note that the presence of this type of benign tumours can mimic HCM.

Finally, heart tumors can mimic an asymmetric HCM. Although signal intensity may be similar between tumoral tissue and normal myocardium in cine imaging, T1 or T2 weighted sequences and LGE may often differentiate between the two and help to delimit these masses (Figure 15).

## Conclusions and key concepts

• CMR can identify the presence and spatial extent of LV hypertrophy, with a better visualization of some segments in comparison to echocardiography. Also, its good spatial resolution allows for accurate thickness measurements.
• The different phenotypic expressions of HCM can be adequately characterized with CMR.
• Myocardial fibrosis (either localized or diffuse) can be identified with CMR. Although several articles and meta-analysis have linked the presence of late gadolinium enhancement with an increased arrhythmic risk, no consensus yet exists.
• CMR can also assess other conditions associated with HCM, such as dynamic left ventricular outflow tract obstruction, mitral regurgitation, diastolic dysfunction, and myocardial ischemia.
• The differential diagnosis of HCM includes other heart pathologies characterized by left ventricle hypertrophy. CMR has a role in the differential diagnosis.

## References

[ref-1] Elliott PM, Anastasakis A, Authors/TaskForce members (2014). ESC Guidelines on diagnosis and management of hypertrophic cardiomyopathy. Eur. Heart J..

[ref-2] Gersh BJ, Maron BJ, Bonow RO (2011). 2011 ACCF/AHA guideline for the diagnosis and treatment of hypertrophic cardiomyopathy: A report of the american college of cardiology foundation/american heart association task force on practice guidelines. Circulation.

[ref-3] Maron MS, Maron BJ, Harrigan C (2009). Hypertrophic cardiomyopathy phenotype revisited after 50 years with cardiovascular magnetic resonance. J. Am. Coll. Cardiol..

[ref-4] Maron BJ, Ommen SR, Semsarian C, Spirito P, Olivotto I, Maron MS (2014). Hypertrophic cardiomyopathy. J. Am. Coll. Cardiol..

[ref-5] Masarone D, Kaski JP, Pacileo G (2018). Epidemiology and clinical aspects of genetic cardiomyopathies. Heart Fail. Clin..

[ref-6] Fourey D, Care M, Siminovitch KA (2017). Prevalence and clinical implication of double mutations in hypertrophic cardiomyopathy: Revisiting the gene-dose effect. Circ. Cardiovasc. Genet..

[ref-7] Sen-Chowdhry S, Jacoby D, Moon JC, McKenna WJ (2016). Update on hypertrophic cardiomyopathy and a guide to the guidelines. Nat. Rev. Cardiol..

[ref-8] Quarta G, Aquaro GD, Pedrotti P (2018). Cardiovascular magnetic resonance imaging in hypertrophic cardiomyopathy: the importance of clinical context. Eur. Heart J. Cardiovasc. Imaging.

[ref-9] Hundley WG, Bluemke DA, Finn JP (2010). ACCF/ACR/AHA/NASCI/SCMR 2010 expert consensus document on cardiovascular magnetic resonance: A report of the american college of cardiology foundation task force on expert consensus documents. Circulation.

[ref-10] Adabag AS, Maron BJ, Appelbaum E (2008). Occurrence and frequency of arrhythmias in hypertrophic cardiomyopathy in relation to delayed enhancement on cardiovascular magnetic resonance. J. Am. Coll. Cardiol..

[ref-11] Moon JC, Messroghli DR, Kellman P (2013). Myocardial T1 mapping and extracellular volume quantification: a Society for Cardiovascular Magnetic Resonance (SCMR) and CMR working group of the European society of cardiology consensus statement. J. Cardiovasc. Magn. Reson..

[ref-12] Ridgway JP (2010). Cardiovascular magnetic resonance physics for clinicians: part I. J. Cardiovasc. Magn. Reson..

[ref-13] Maron MS, Maron BJ (2015). Clinical impact of contemporary cardiovascular magnetic resonance imaging in hypertrophic cardiomyopathy. Circulation.

[ref-14] Patel AR, Kramer CM (2017). Role of cardiac magnetic resonance in the diagnosis and prognosis of nonischemic cardiomyopathy. JACC. Cardiovasc. Imaging.

[ref-15] Webb J, Villa A, Bekri I (2017). Usefulness of cardiac magnetic resonance imaging to measure left ventricular wall thickness for determining risk scores for sudden cardiac death in patients with hypertrophic cardiomyopathy. Am. J. Cardiol..

[ref-16] Bois JP, Geske JB, Foley TA, Ommen SR, Pellikka PA (2017). Comparison of maximal wall thickness in hypertrophic cardiomyopathy differs between magnetic resonance imaging and transthoracic echocardiography. Am. J. Cardiol..

[ref-17] Posma JL, Blanksma PK, van der Wall EE, Hamer HP, Mooyaart EL, Lie KI (1996). Assessment of quantitative hypertrophy scores in hypertrophic cardiomyopathy: magnetic resonance imaging versus echocardiography. Am. Heart J..

[ref-18] Pons-Lladó G, Carreras F, Borrás X, Palmer J, Llauger J, Bayés de Luna A (1997). Comparison of morphologic assessment of hypertrophic cardiomyopathy by magnetic resonance versus echocardiographic imaging. Am. J. Cardiol..

[ref-19] Lorca R, Gómez J, Martín M (2018). Insights into hypertrophic cardiomyopathy evaluation through follow-up of a founder pathogenic variant. Rev. Esp. Cardiol. (Engl. Ed)..

[ref-20] Cresti A, Cannarile P, Aldi E (2018). Multimodality imaging and clinical significance of congenital ventricular outpouchings: Recesses, diverticula, aneurysms, clefts, and crypts. J. Cardiovasc. Echogr..

[ref-21] Harrigan CJ, Appelbaum E, Maron BJ (2008). Significance of papillary muscle abnormalities identified by cardiovascular magnetic resonance in hypertrophic cardiomyopathy. Am. J. Cardiol..

[ref-22] Bohl S, Wassmuth R, Abdel-Aty H (2008). Delayed enhancement cardiac magnetic resonance imaging reveals typical patterns of myocardial injury in patients with various forms of non-ischemic heart disease. Int. J. Cardiovasc. Imaging.

[ref-23] Kato S, Nakamori S, Bellm S (2016). Myocardial native T1 time in patients with hypertrophic cardiomyopathy. Am. J. Cardiol..

[ref-24] Everett RJ, Stirrat CG, Semple SIR, Newby DE, Dweck MR, Mirsadraee S (2016). Assessment of myocardial fibrosis with T1 mapping MRI. Clin. Radiol..

[ref-25] Ennis DB, Epstein FH, Kellman P, Fananapazir L, McVeigh ER, Arai AE (2003). Assessment of regional systolic and diastolic dysfunction in familial hypertrophic cardiomyopathy using MR tagging. Magn. Reson. Med..

[ref-26] van Dockum WG, Kuijer JPA, Götte MJW (2006). Septal ablation in hypertrophic obstructive cardiomyopathy improves systolic myocardial function in the lateral (free) wall: a follow-up study using CMR tissue tagging and 3D strain analysis. Eur. Heart J..

[ref-27] Maron MS, Olivotto I, Harrigan C (2011). Mitral valve abnormalities identified by cardiovascular magnetic resonance represent a primary phenotypic expression of hypertrophic cardiomyopathy. Circulation.

[ref-28] Florian A, Masci PG, De Buck S (2012). Geometric assessment of asymmetric septal hypertrophic cardiomyopathy by CMR. JACC. Cardiovasc. Imaging.

[ref-29] Marchesini M, Uguccioni L, Parisi R The role of cardiac magnetic resonance imaging in hypertrophic cardiomyopathy. Rev. Cardiovasc. Med..

[ref-30] Hayat U, Lim C, Chen S (2016). Hourglass appearance on ventriculography: insights from cardiac magnetic resonance imaging. Heart.

[ref-31] Fattori R, Biagini E, Lorenzini M, Buttazzi K, Lovato L, Rapezzi C (2010). Significance of magnetic resonance imaging in apical hypertrophic cardiomyopathy. Am. J. Cardiol..

[ref-32] Moon JCC, Fisher NG, McKenna WJ, Pennell DJ (2004). Detection of apical hypertrophic cardiomyopathy by cardiovascular magnetic resonance in patients with non-diagnostic echocardiography. Heart.

[ref-33] Kwon DH, Setser RM, Popović ZB (2008). Association of myocardial fibrosis, electrocardiography and ventricular tachyarrhythmia in hypertrophic cardiomyopathy: a delayed contrast enhanced MRI study. Int. J. Cardiovasc. Imaging.

[ref-34] Maron BJ (2009). Distinguishing hypertrophic cardiomyopathy from athlete’s heart physiological remodelling: clinical significance, diagnostic strategies and implications for preparticipation screening. Br. J. Sports Med..

[ref-35] Hansen MW, Merchant N (2007). MRI of hypertrophic cardiomyopathy: part 2, Differential diagnosis, risk stratification, and posttreatment MRI appearances. AJR. Am. J. Roentgenol..

[ref-36] Keeling AN, Carr JC, Choudhury L (2010). Right ventricular hypertrophy and scarring in mutation positive hypertrophic cardiomyopathy. Eur. Heart J..

[ref-37] Maron MS, Hauser TH, Dubrow E (2007). Right ventricular involvement in hypertrophic cardiomyopathy. Am. J. Cardiol..

[ref-38] Cheng S, Choe YH, Ota H (2018). CMR assessment and clinical outcomes of hypertrophic cardiomyopathy with or without ventricular remodeling in the end-stage phase. Int. J. Cardiovasc. Imaging.

[ref-39] Kwon DH, Setser RM, Thamilarasan M (2008). Abnormal papillary muscle morphology is independently associated with increased left ventricular outflow tract obstruction in hypertrophic cardiomyopathy. Heart.

[ref-40] Cheng S, Fang M, Cui C (2018). LGE-CMR-derived texture features reflect poor prognosis in hypertrophic cardiomyopathy patients with systolic dysfunction: preliminary results. Eur. Radiol..

[ref-41] Conte MR, Bongioanni S, Chiribiri A (2011). Late gadolinium enhancement on cardiac magnetic resonance and phenotypic expression in hypertrophic cardiomyopathy. Am. Heart J..

[ref-42] Axelsson Raja A, Farhad H, Valente AM (2018). Prevalence and progression of late gadolinium enhancement in children and adolescents with hypertrophic cardiomyopathy. Circulation.

[ref-43] Rudolph A, Abdel-Aty H, Bohl S (2009). Noninvasive detection of fibrosis applying contrast-enhanced cardiac magnetic resonance in different forms of left ventricular hypertrophy relation to remodeling. J. Am. Coll. Cardiol..

[ref-44] Maron MS (2012). Clinical utility of cardiovascular magnetic resonance in hypertrophic cardiomyopathy. J. Cardiovasc. Magn. Reson..

[ref-45] Moon JCC, Reed E, Sheppard MN (2004). The histologic basis of late gadolinium enhancement cardiovascular magnetic resonance in hypertrophic cardiomyopathy. J. Am. Coll. Cardiol..

[ref-46] Ho CY, López B, Coelho-Filho OR (2010). Myocardial fibrosis as an early manifestation of hypertrophic cardiomyopathy. N. Engl. J. Med..

[ref-47] Leonardi S, Raineri C, De Ferrari GM (2009). Usefulness of cardiac magnetic resonance in assessing the risk of ventricular arrhythmias and sudden death in patients with hypertrophic cardiomyopathy. Eur. Heart J..

[ref-48] Suksaranjit P, McGann CJ, Akoum N (2016). Prognostic implications of left ventricular scar determined by late gadolinium enhanced cardiac magnetic resonance in patients with atrial fibrillation. Am. J. Cardiol..

[ref-49] Bruder O, Wagner A, Jensen CJ (2010). Myocardial scar visualized by cardiovascular magnetic resonance imaging predicts major adverse events in patients with hypertrophic cardiomyopathy. J. Am. Coll. Cardiol..

[ref-50] O’Hanlon R, Grasso A, Roughton M (2010). Prognostic significance of myocardial fibrosis in hypertrophic cardiomyopathy. J. Am. Coll. Cardiol..

[ref-51] Suk T, Edwards C, Hart H, Christiansen JP (2008). Myocardial scar detected by contrast-enhanced cardiac magnetic resonance imaging is associated with ventricular tachycardia in hypertrophic cardiomyopathy patients. Hear. Lung Circ..

[ref-52] Maron MS, Appelbaum E, Harrigan CJ (2008). Clinical profile and significance of delayed enhancement in hypertrophic cardiomyopathy. Circ. Heart Fail..

[ref-53] Rubinshtein R, Glockner JF, Ommen SR (2010). Characteristics and clinical significance of late gadolinium enhancement by contrast-enhanced magnetic resonance imaging in patients with hypertrophic cardiomyopathy. Circ. Heart Fail..

[ref-54] Smith BM, Dorfman AL, Yu S (2014). Clinical significance of late gadolinium enhancement in patients. Am. J. Cardiol..

[ref-55] Hen Y, Iguchi N, Utanohara Y (2014). Prognostic value of late gadolinium enhancement on cardiac magnetic resonance imaging in Japanese hypertrophic cardiomyopathy patients. Circ. J..

[ref-56] Ismail TF, Jabbour A, Gulati A (2014). Role of late gadolinium enhancement cardiovascular magnetic resonance in the risk stratification of hypertrophic cardiomyopathy. Heart.

[ref-57] Klopotowski M, Kukula K, Malek LA (2016). The value of cardiac magnetic resonance and distribution of late gadolinium enhancement for risk stratification of sudden cardiac death in patients with hypertrophic cardiomyopathy. J. Cardiol..

[ref-58] Chan RH, Maron BJ, Olivotto I (2015). Significance of late gadolinium enhancement at right ventricular attachment to ventricular septum in patients with hypertrophic cardiomyopathy. Am. J. Cardiol..

[ref-59] Green JJ, Berger JS, Kramer CM, Salerno M (2012). Prognostic value of late gadolinium enhancement in clinical outcomes for hypertrophic cardiomyopathy. JACC. Cardiovasc. Imaging.

[ref-60] Briasoulis A, Mallikethi-Reddy S, Palla M, Alesh I, Afonso L (2015). Myocardial fibrosis on cardiac magnetic resonance and cardiac outcomes in hypertrophic cardiomyopathy: a meta-analysis. Heart.

[ref-61] Weng Z, Yao J, Chan RH (2016). Prognostic value of LGE-CMR in HCM: A meta-analysis. JACC. Cardiovasc. Imaging.

[ref-62] He D, Ye M, Zhang L, Jiang B (2018). Prognostic significance of late gadolinium enhancement on cardiac magnetic resonance in patients with hypertrophic cardiomyopathy. Heart Lung.

[ref-63] Hinojar R, Varma N, Child N (2015). T1 mapping in discrimination of hypertrophic phenotypes: hypertensive heart disease and hypertrophic cardiomyopathy: Findings from the international T1 multicenter cardiovascular magnetic resonance study. Circ. Cardiovasc. Imaging.

[ref-64] Karur GR, Robison S, Iwanochko RM (2018). Use of myocardial T1 mapping at 3.0 T to differentiate anderson-fabry disease from hypertrophic cardiomyopathy. Radiology.

[ref-65] Martinez-Naharro A, Treibel TA, Abdel-Gadir A (2017). Magnetic resonance in transthyretin cardiac amyloidosis. J. Am. Coll. Cardiol..

[ref-66] Ho CY, Abbasi SA, Neilan TG (2013). T1 measurements identify extracellular volume expansion in hypertrophic cardiomyopathy sarcomere mutation carriers with and without left ventricular hypertrophy. Circ. Cardiovasc. Imaging.

[ref-67] Swoboda PP, McDiarmid AK, Erhayiem B (2016). Assessing myocardial extracellular volume by T1 mapping to distinguish hypertrophic cardiomyopathy from athlete’s heart. J. Am. Coll. Cardiol..

[ref-68] Parekh K, Markl M, Deng J, de Freitas RA, Rigsby CK (2017). T1 mapping in children and young adults with hypertrophic cardiomyopathy. Int. J. Cardiovasc. Imaging.

[ref-69] Swoboda PP, McDiarmid AK, Erhayiem B (2017). Effect of cellular and extracellular pathology assessed by T1 mapping on regional contractile function in hypertrophic cardiomyopathy. J. Cardiovasc. Magn. Reson..

[ref-70] Avanesov M, Münch J, Weinrich J (2017). Prediction of the estimated 5-year risk of sudden cardiac death and syncope or non-sustained ventricular tachycardia in patients with hypertrophic cardiomyopathy using late gadolinium enhancement and extracellular volume CMR. Eur. Radiol..

[ref-71] Patel P, Dhillon A, Popovic ZB (2015). Left ventricular outflow tract obstruction in hypertrophic cardiomyopathy patients without severe septal hypertrophy: implications of mitral valve and papillary muscle abnormalities assessed using cardiac magnetic resonance and echocardiography. Circ. Cardiovasc. Imaging.

[ref-72] Maron BJ, Haas TS, Maron MS (2014). Left atrial remodeling in hypertrophic cardiomyopathy and susceptibility markers for atrial fibrillation identified by cardiovascular magnetic resonance. Am. J. Cardiol..

[ref-73] Zhu Y, Park E-A, Lee W (2015). Extent of late gadolinium enhancement at right ventricular insertion points in patients with hypertrophic cardiomyopathy: relation with diastolic dysfunction. Eur. Radiol..

[ref-74] Hinojar R, Fernández-Golfín C, González-Gómez A (2017). Prognostic implications of global myocardial mechanics in hypertrophic cardiomyopathy by cardiovascular magnetic resonance feature tracking. Relations to left ventricular hypertrophy and fibrosis. Int. J. Cardiol..

[ref-75] Schuster A, Morton G, Hussain ST (2013). The intra-observer reproducibility of cardiovascular magnetic resonance myocardial feature tracking strain assessment is independent of field strength. Eur. J. Radiol..

[ref-76] Pelliccia A, Maron MS, Maron BJ (2012). Assessment of left ventricular hypertrophy in a trained athlete: differential diagnosis of physiologic athlete’s heart from pathologic hypertrophy. Prog. Cardiovasc. Dis..

[ref-77] Petersen SE, Selvanayagam JB, Francis JM (2005). Differentiation of athlete’s heart from pathological forms of cardiac hypertrophy by means of geometric indices derived from cardiovascular magnetic resonance. J. Cardiovasc. Magn. Reson..

[ref-78] Rodrigues JCL, Rohan S, Ghosh Dastidar A (2017). Hypertensive heart disease versus hypertrophic cardiomyopathy: multi-parametric cardiovascular magnetic resonance discriminators when end-diastolic wall thickness ≥ 15 mm. Eur. Radiol..

[ref-79] D’souza RS, Levandowski C, Slavov D (2014). Danon disease: clinical features, evaluation, and management. Circ. Heart Fail..

